# Salivary peptidome analysis and protease prediction during orthodontic treatment with fixed appliances

**DOI:** 10.1038/s41598-022-26969-3

**Published:** 2023-01-12

**Authors:** Fidaa Wazwaz, Hayder Saloom, Jack W. Houghton, Martyn T. Cobourne, Guy H. Carpenter

**Affiliations:** 1 Department of Orthodontics, Faculty of Dentistry, Oral & Craniofacial Sciences, Centre for Craniofacial Development & Regeneration, King’s College London, London, UK; 2 Salivary Biology, Centre for Host-Microbiome Interactions, Faculty of Dentistry, Oral & Craniofacial Sciences, King’s College London, London, UK; 3 Orthodontic Department, College of Dentistry, University of Baghdad, Baghdad, Iraq; 4 Department of Chemistry, Molecular Sciences Research Hub, Imperial College London, London, UK; 5 Department of Orthodontics, Faculty of Dentistry, Oral & Craniofacial Sciences, Centre for Craniofacial Development & Regeneration, King’s College London, Guy’s Hospital, London, SE1 9RT UK

**Keywords:** Biomarkers, Health care, Dentistry

## Abstract

Orthodontic tooth movement (OTM) occurs through proteolytic remodelling within the periodontium following the application of external force to the tooth. This study describes the first characterization of the salivary peptidome and protease profile during the alignment stage of fixed appliance orthodontic treatment. Unstimulated whole mouth saliva from 16 orthodontic patients (10 males, 6 females, mean (SD) age 15.2 (1.6) years) was collected prior to fixed appliance placement (T1), 1-h (T2), 1-week (T3) following fixed appliance placement and on completion of mandibular arch alignment (T4). Salivary peptides were extracted using filtration followed by mass spectrometry to identify amino acid sequences. Protease prediction was carried out in silico using Proteasix and validated with gelatin zymography and enzyme-linked immunosorbent assay. A total of 2852 naturally-occurring peptides were detected, originating from 436 different proteins. Both collagen and statherin-derived peptide levels were increased at T2. Proteasix predicted 73 proteases potentially involved in generating these peptides, including metalloproteinases, calpains and cathepsins. Changes in predicted activity of proteases over time were also observed, with most metalloproteinases showing increased predicted activity at T2–T3. Increased gelatinolytic activity and MMP8/MMP9 levels were detected at T3. Collectively, multiple protein targets and changes in protease-predicted activity during OTM have been identified.

## Introduction

Orthodontic tooth movement (OTM) provides the basis of orthodontic treatment and involves co-ordinated tissue remodelling in the periodontal ligament and alveolar bone following force application to the tooth^1^. Force transduction within the periodontium triggers a localised aseptic inflammation mediated by cytokines, prostaglandins, tissue necrosis factors and proteases, followed by degradation or synthesis of extracellular matrix (ECM) in the periodontal ligament and alveolar bone. These processes, in turn promote the secretion of many proteins and enzymes into the gingival crevicular fluid (GCF) and whole mouth saliva (WMS)^1,2^.

Several enzymes have been identified in WMS, including amylase, carbonic anhydrase, catalase, and proteases^3^. Proteases are one of the largest families, with more than 500 encoded in the human genome^4^. Initiation, progression, and resolution of inflammation, as well as ECM remodelling are regulated by proteases^5^. Cathepsins and matrix metalloproteinases (MMPs) are involved in remodelling of periodontal ligament, ECM and alveolar bone^1^, in particular GCF MMP1–3, MMP8–9, MMP13, MMP14^2,6–9^ and WMS MMP8–9, MMP12 concentrations all increasing during OTM^10^.

WMS is a complex fluid that contains secretions from all the major and minor salivary glands, constituents from GCF, the oral microbiome and dietary inputs. More recently, proteomic and peptidomic non-targeted approaches of WMS have provided biomarkers for the early detection, progression, monitoring, and responses to many oral and systemic disorders treatments^11,12^. In silico prediction of proteases using the open-source software Proteasix (www.proteasix.org) has predicted proteases potentially involved in the generation of the peptides in patients with periodontitis^13^, wound infection^14^, diabetic nephropathy^15^ and cardiorenal syndrome^16^. Proteasix uses information about naturally-occurring peptides corresponding to the protein using the UniProt ID as identified by mass spectrometry to predict potential cleaving proteases. Retrieving information about cleavage sites from protease databases (MEROPS, BRENDA) allows the generation of a list of predicted proteases^17^. The analysis of cleavage site-specificity by Proteasix can predict multiple proteases from a single sample revealing the proteolytic events underlying physiological and pathological processes taking place within the oral cavity^18^.

To our knowledge, the WMS natural peptidome generated during OTM has not previously been investigated. This retrospective longitudinal study has used a peptidomic approach, supplemented with mass spectrometry and bioinformatics to predict the profile and activity pattern of proteases responsible for WMS peptide generation. Susceptible protein targets have been identified, and time-dependent changes in the salivary peptidome and predicted proteases have been assessed during the alignment stage of orthodontic treatment with fixed appliances. Further targeted approaches used zymography and ELISA to validate the prediction results.

## Results

This study investigated WMS from 16 participants (10 male, 6 female) with a mean (SD) age of 15.2 (1.6) years and a mean (SD) crowding of 6.4 (2.2) mm, with only three participants having extraction-based treatment. Mean plaque index was: T1 0.57 (0.3); T3 0.76 (0.32); T4 1.09 (0.40); whilst gingival index was T1 0.73 (0.31); T3 0.84 (0.22); T4 1.25 (0.35). Plaque and gingival indices were increased significantly at T4 compared with T1 (*p* < 0.001). Mean WMS flow-rate (ml/min) was: T1 0.67 (0.29); T2 0.85 (0.37); T3 1.03 (0.45); T4 0.93 (0.37). In addition, mean protein content (mg/ml) was: T1 1.51 (0.48); T2 1.55 (0.34); T3 1.65 (0.68); T4 1.14 (0.48). No statistically significant differences were detected in either measurement between time-points (assessed using repeated measures ANOVA).

### Peptidome characteristics of WMS

**Figure 1:**
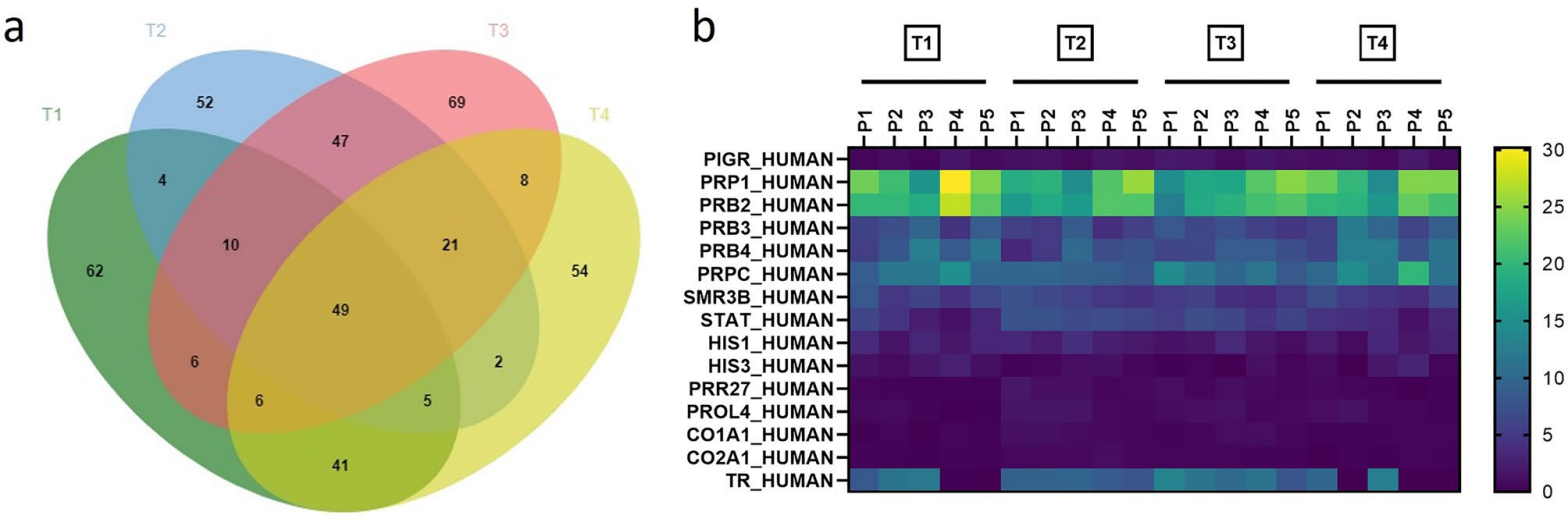
(**a**) Venn diagram showing the distribution of proteins of origin of the identified peptides by mass spectrometry at four time-points. *T1* baseline (before placement of orthodontic appliance), *T2* 1 h after placement of orthodontic appliance, *T3* 1 week after placement of orthodontic appliance, *T4* end of the alignment. (**b**) Heatmap displaying the percentages of peptides for the most abundant common proteins among all the participants (p1, p2, p3, p4, and p5) at four time-points. The scale refers to the percentages of peptides of each protein in the total number of peptides for each participant.

A total of 2852 naturally-occurring peptides were identified by mass spectrometry originating from 436 different proteins with 49 common to all time-points (Fig. 1a). Percentage of peptides identified for each common protein was calculated (Fig. 1b) (Supplementary Appendix Table S1). The most abundant belonged to the major salivary proteins, mainly proline-rich proteins, statherin, histatins and P-B peptide. When the percentages of peptides identified for each common protein were compared over time, no statistically significant changes were found for PIGR, PRP1, PRB2, PRB3, PRBC, SMR3B, HIS1 at all time-points. Statistically significant degradation of STAT, PROL4, CO1A1 (all p < 0.05) and CO2A1 (p < 0.01) were observed at T2 compared with T1, with this degradation returning to baseline levels at T4. Peptides belonging to PRR27 were significantly increased at T2 and T3 (both p < 0.05), also returning to baseline at T4. Conversely, the percentages of peptides belonging to PRB4 were significantly decreased at T2 (p < 0.01), returning to T1 levels by T4. Percentages of peptides belonging to HIS3 were significantly decreased at both T2 (p < 0.01) and T3 (p < 0.05) (Supplementary Appendix Fig. S1).

### Prediction of protease activity

**Figure 2:**
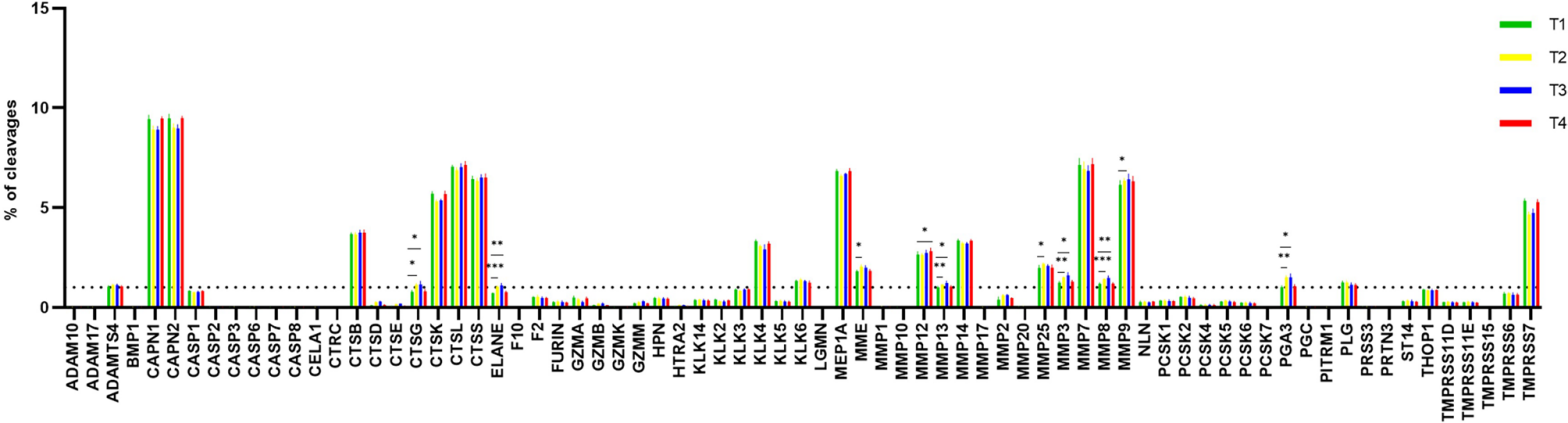
Graph showing the profile of all proteases as predicted by Proteasix. The bars represent the percentage of cleavages for each predicted protease at four time-points. *T1* baseline (before placement of orthodontic appliance), *T2* 1 h after placement of orthodontic appliance, *T3* 1 week after placement of orthodontic appliance, *T4* end of the alignment. The interrupted line represents a percentage threshold (1%) to consider the activity of the proteases. Data are shown as mean ± SD. Data were analysed by repeated measures ANOVA; *p < 0.05; **p < 0.01; ***p < 0.001.

The profile of all predicted proteases is shown in Fig. 2. In total, 73 were predicted to be active in WMS of participants (Table 1). For each protease, the percentage of cleavage from total cleavage events for each participant was calculated and a percentage threshold of cleavage set at 1% to consider activity of a particular protease (Fig. 2)^16^. Twenty-four proteases had a percentage threshold of cleavage > 1% and amongst these, calpains, MMPs and cathepsins were the most prevalent groups and potentially implicated in the generation of WMS peptides at all time-points. When the predicted activity of these proteases was compared over time, there was a statistically significant increase of CTSG (cathepsin G) (p < 0.05, p < 0.05), ELANE (neutrophil elastase) (p < 0.001, p < 0.01), MMP13 (p < 0.01, p < 0.05), MMP3 (p < 0.01, p < 0.05), MMP8 (p < 0.001, p < 0.01), PGA3 (pepsin) (p < 0.01, p < 0.05) at both T2 and T3 compared to T1. Predicted activity of MME (neprilysin) (p < 0.05), MMP25 (p < 0.05), MMP9 (p < 0.05) was significantly increased at T2, whereas MMP12 (p < 0.05) predicted activity was significantly increased at T4 compared to T1. In contrast, predicted activity of CAPN1 (p < 0.001, p < 0.001), CAPN2 (p < 0.001, p < 0.001), CTSK (cathepsin K) (p < 0.01, p < 0.05), MEP1A (p < 0.01, p < 0.05), TMPRSS7 (p < 0.01, p < 0.05) was significantly decreased at both T2 and T3 compared to T1. Furthermore, predicted activity of MMP7 (p < 0.01), KLK4 (kallikrein 4) (p < 0.05) was significantly decreased at T3 and T2. No changes in level of predicted activity of ADAMTS4, CTSB (cathepsin B), CTSL (cathepsin L), CTSS (cathepsin S), KLK6 (kallikrein 4), MMP14, PLG (plasminogen) were found at any time-points.

**Table 1:** List of all proteases (symbols, accession numbers, and names) predicted by Proteasix.

Protease symbol	Accession no	Protease name
ADAM10	O14672	Disintegrin and metalloproteinase domain-containing protein 10
ADAM17	P78536	Disintegrin and metalloproteinase domain-containing protein 17
ADAMTS4	O75173	A disintegrin and metalloproteinase with thrombospondin motifs 4
BMP1	P13497	Bone morphogenetic protein 1
CAPN1	P07384	Calpain-1 catalytic subunit
CAPN2	P17655	Calpain-2 catalytic subunit
CASP1	P29466	Caspase-1
CASP2	P42575	Caspase-2
CASP3	P42574	Caspase-3
CASP6	P55212	Caspase-6
CASP7	P55210	Caspase-7
CASP8	Q14790	Caspase-8
CELA1	Q9UNI1	Chymotrypsin-like elastase family member 1
CTRC	Q99895	Chymotrypsin-C
CTSB	P07858	Cathepsin B
CTSD	P07339	Cathepsin D
CTSE	P14091	Cathepsin E
CTSG	P08311	Cathepsin G
CTSK	P43235	Cathepsin K
CTSL	P07711	Cathepsin L1
CTSS	P25774	Cathepsin S
ELANE	P08246	Neutrophil elastase
F10	P00742	Coagulation factor X
F2	P00734	Prothrombin
FURIN	P09958	Furin
GZMA	P12544	Granzyme A
GZMB	P10144	Granzyme B
GZMK	P49863	Granzyme K
GZMM	P51124	Granzyme M
HPN	P05981	Serine protease hepsin
HTRA2	O43464	Serine protease HTRA2, mitochondrial
KLK14	Q9P0G3	Kallikrein-14
KLK2	P20151	Kallikrein-2
KLK3	P07288	Prostate-specific antigen
KLK4	Q9Y5K2	Kallikrein-4
KLK5	Q9Y337	Kallikrein-5
KLK6	Q92876	Kallikrein-6
LGMN	Q99538	Legumain
MEP1A	Q16819	Meprin A subunit alpha
MME	P08473	Neprilysin
MMP1	P03956	Interstitial collagenase
MMP10	P09238	Stromelysin-2
MMP12	P39900	Macrophage metalloelastase
MMP13	P45452	Collagenase 3
MMP14	P50281	Matrix metalloproteinase-14
MMP17	Q9ULZ9	Matrix metalloproteinase-17
MMP2	P08253	72 kDa type IV collagenase
MMP20	O60882	Matrix metalloproteinase-20
MMP25	Q9NPA2	Matrix metalloproteinase-25
MMP3	P08254	Stromelysin-1
MMP7	P09237	Matrilysin
MMP8	P22894	Neutrophil collagenase
MMP9	P14780	Matrix metalloproteinase-9
NLN	Q9BYT8	Neurolysin, mitochondrial
PCSK1	P29120	Neuroendocrine convertase 1
PCSK2	P16519	Neuroendocrine convertase 2
PCSK4	Q6UW60	Proprotein convertase subtilisin/kexin type 4
PCSK5	Q92824	Proprotein convertase subtilisin/kexin type 5
PCSK6	P29122	Proprotein convertase subtilisin/kexin type 6
PCSK7	Q16549	Proprotein convertase subtilisin/kexin type 7
PGA3	P0DJD8	Pepsin A-3
PGC	P20142	Gastricsin
PITRM1	Q5JRX3	Presequence protease, mitochondrial
PLG	P00747	Plasminogen
PRSS3	P35030	Trypsin-3
PRTN3	P24158	Myeloblastin
ST14	Q9Y5Y6	Suppressor of tumorigenicity 14 protein
THOP1	P52888	Thimet oligopeptidase
TMPRSS11D	O60235	Transmembrane protease serine 11D
TMPRSS11E	Q9UL52	Transmembrane protease serine 11E
TMPRSS15	P98073	Enteropeptidase
TMPRSS6	Q8IU80	Transmembrane protease serine 6
TMPRSS7	Q7RTY8	Transmembrane protease serine 7

### Targeted approaches to validate proteases predictions

**Figure 3:**
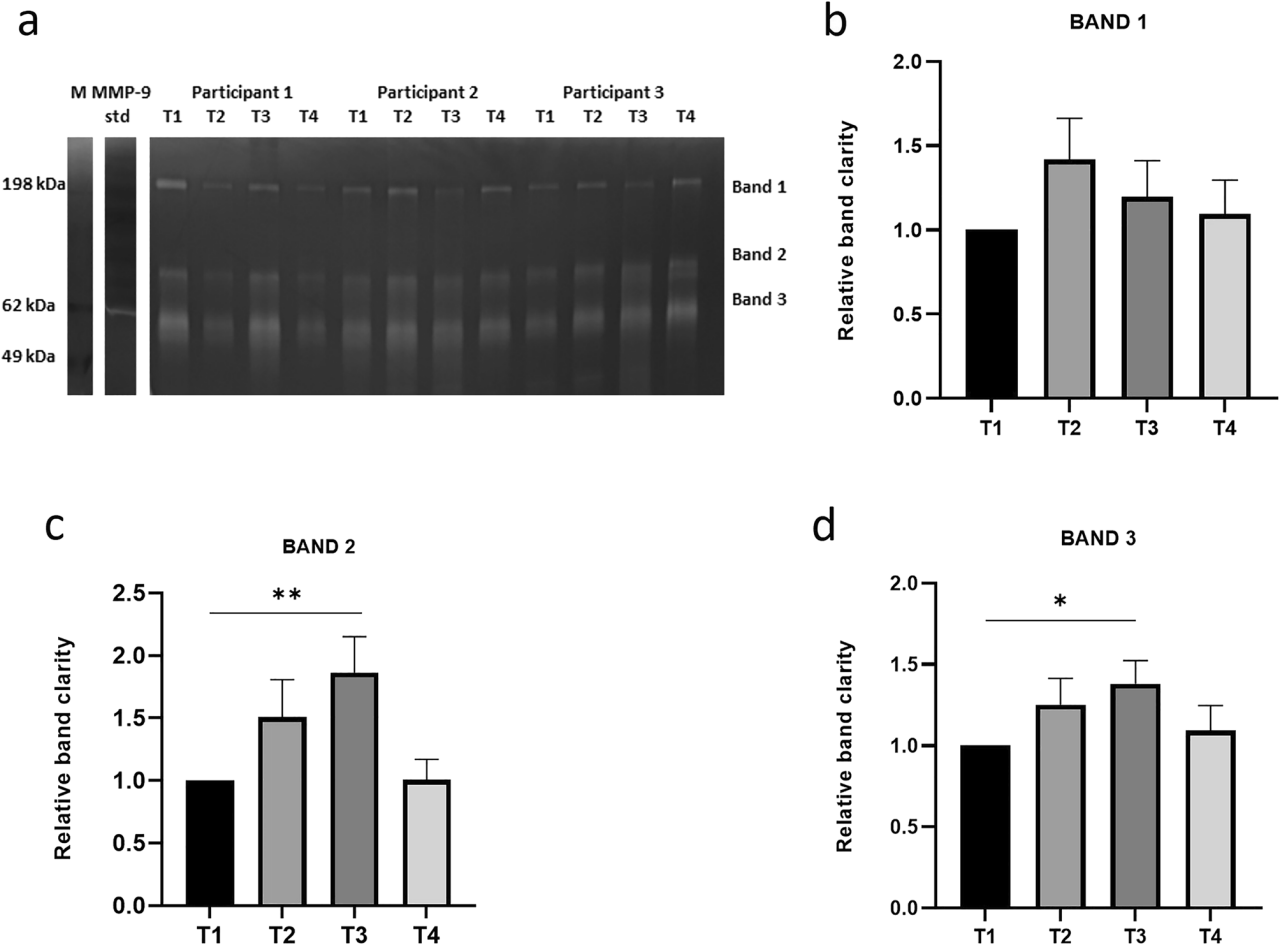
Gelatin zymography for the investigation of gelatinolytic activity in unstimulated whole mouth saliva of 16 participants at four time-points. (**a**) Representative example of Coomassie-stained zymogram gel demonstrating bands with gelatinolytic activity at three different molecular weights (uncropped gel with original arrangement of lanes is presented in Supplementary Appendix Fig. S2). (**b–d**) Relative quantification of band intensity of bands 1, 2, and 3. The fold change of gelatinolytic activity at T2, T3, and T4 for each band was assessed relative to T1. *T1* baseline (before placement of orthodontic appliance), *T2* 1 h after placement of orthodontic appliance, *T3* 1 week after placement of orthodontic appliance, *T4* end of the alignment, *M* molecular weight markers, *MMP-9 std* matrix metalloproteinase-9 standard; *p < 0.05; **p < 0.01.

**Figure 4:**
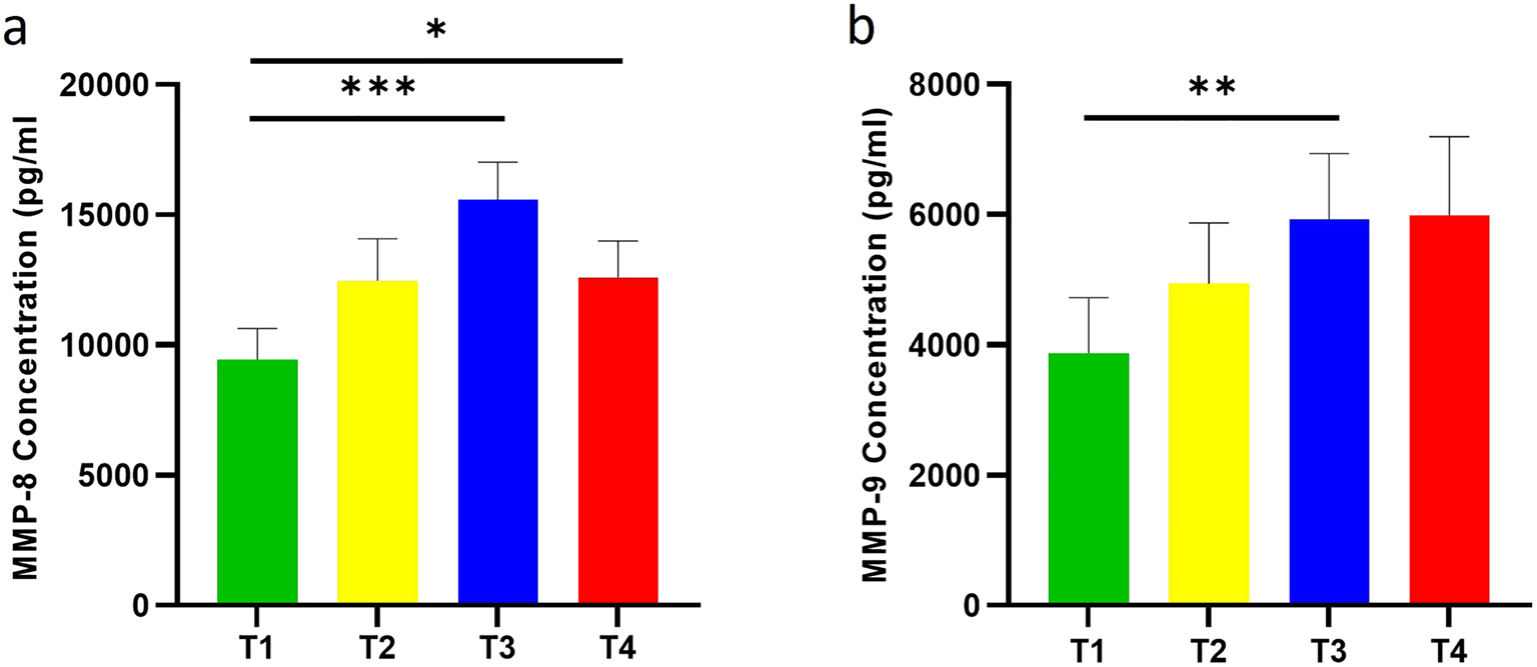
Graphs showing the levels of matrix metalloproteinase-8 (MMP8) (**a**) and matrix metalloproteinase-9 (MMP9) (**b**), as measured by ELISA (Enzyme-linked immunosorbent assay), in unstimulated whole mouth saliva of 16 participants at four time-points. *T1* baseline (before placement of orthodontic appliance), *T2* 1 h after placement of orthodontic appliance, *T3* 1 week after placement of orthodontic appliance *T4* end of the alignment. Data were analysed by repeated measures ANOVA; *p < 0.05; **p < 0.01; ***p < 0.001.

WMS from all 16 participants was used with gelatin zymography to directly assess gelatinolytic activity and with ELISA to assess protease abundance. Three distinct bands were identified in all samples at approximately 190 kDa (band 1), 72 kDa (band 2) and 62 kDa (band 3) (Fig. 3a) (Supplementary Appendix Fig. S2). Gelatinolytic activity increased over time and returned to baseline levels at T4, being around 1.8× more elevated at T3 than T1 for band 2 (p < 0.01) and 1.4× more at T3 than T1 for band 3 (p < 0.05) (Fig. 3b–d). ELISA demonstrated that MMP8 levels increased over time but were only statistically significant at T3 and T4 compared with T1 (p < 0.001; p < 0.05, respectively) (Fig. 4a). MMP9 levels were significantly increased at T3 compared with T1 (p < 0.01) (Fig. 4b).

## Discussion

This study describes the first characterisation of the salivary peptidome and protease profile during OTM using a peptidomic approach aided by mass spectrometry and bioinformatics. Proteolytic activity plays a crucial role in ECM remodelling and several enzymes have been implicated including serine, aspartate, and cysteine proteases^19^. Our non-targeted approach identified 73 predicted proteases responsible for producing peptides of which only 57 matched to a previous study of WMS using the same software^13^. The additional 16 novel proteases could be attributed to changes in the Proteasix algorithm since the original publication^4^, age-related differences in the WMS proteome and peptidome^20^ or more likely the effect of OTM. From the 73, 24 had > 1% cleavage^16^ with calpains, MMPs, cathepsins the most prevalent groups.

OTM occurs through remodelling of the periodontium mediated by acute inflammation characterized by vascular changes, leukocyte^1,8,19^ and neutrophil infiltration^21^ as early as 1-h following the application of orthodontic force^22^. Thereby, the time-points used in this study were chosen in an attempt to observe the different biological changes that underpin OTM^23^. A statistically significant increase in predicted activity of CTSG, ELANE, PGA3 was found at 1-h and 1-week following appliance placement. Neutrophil azurophilic granules contain ELANE and CTSG, which regulate inflammation and modulation of the immune response^14,24^ through retention of pro- and anti-inflammatory activities^21^. These proteases have not previously been investigated in relation to OTM; however, they seem to have an important role in regulation of the acute inflammatory reaction during initial movement.

MMPs play a pivotal role in ECM remodelling and are involved in inflammatory regulation^19^ and MMP8, MMP9 and MMP13 are produced by polymorphonuclear leukocytes^8,24,25^. MMPs have been extensively investigated in GCF and to a lesser extent WMS, during OTM. Our results reveal significantly increased predicted activity of MMP3, MMP8, MMP13 at 1-h and 1-week, whilst MMP9, MMP25, MME were significantly increased 1-h after appliance placement and MMP12 only at the end of alignment. These predictions were validated by ELISA, which confirmed MMP8 levels were significantly increased at 1-week until end of alignment, whereas MMP9 was significantly increased only at 1-week. In contrast to the predicted results, no statistically significant difference was identified in MMP8 or MMP9 levels 1-h after appliance placement when assessed by ELISA possibly because ELISA detects total MMPs (both pro- and active forms)^26^ and actual activity might be masked by total protein measurement. Notably, WMS MMP8, MMP9, MMP12 levels increased at 1-h^10^ and 1-week^27^ and GCF MMP3, MMP8, MMP9, MMP13 levels have previously positively correlated with OTM^2,6–9^. It has also been shown that orthodontic force application significantly increases MMP8^8^, MMP3, MMP13, and MMP9 levels^28^ in GCF 1-h after orthodontic appliance activation, which agrees with our predicted results.

MME has never previously been studied in OTM; however, one previous study reported that MME mRNA levels were upregulated in periodontitis-affected gingival tissues compared with healthy tissues and that MME expression was detected in neutrophils and fibroblasts in those tissues. In addition, they reported that MME contributes to the regulation of inflammation by degrading IL-1β, which is a crucial inflammatory cytokine^29^. Therefore, MME may have a role in the aseptic inflammatory reaction associated with OTM. Additionally, our predicted results showed that MMP7 activity was significantly decreased 1-week after appliance placement, which contradicts previous data^30,31^.

Our results showed that CAPN1, CAPN2, MEP1A, TMPRSS7 and KLK4 predicted activity was significantly decreased during OTM however, no information is available concerning the production of these proteases during this process. There are few studies on the role of cathepsin during OTM in humans whilst in rats OTM induced a statistically significant increase in CTSK gene expression^32^. Periodontal cells express CTSB (cathepsin B) but not CTSK^33^; Previous studies reported contradictory results on the level of CTSB in GCF; one study reported on an increase in the level of CTSB after 24-h but no change after 1-h and 1-week of OTM^33^ and the second study reported on a decrease in the level of CTSB after 24-h^34^. Therefore, further investigations are necessary to establish the link between cathepsins and OTM in humans.

Proteolysis is the primary source of peptides and significant efforts have been made to identify resultant fragments, cleavage sites and implicated proteases. The most abundant peptides belonged to the major salivary proteins, mainly proline-rich statherin, histatins and P-B peptide^25,35^. The ECM of soft and hard periodontal tissues is comprised mainly of type I collagen. Collagen breakdown is considered critical for periodontal and alveolar bone remodelling associated with OTM^36–38^ and total type I collagenase activity in GCF is increased 10-times in GCF of orthodontic patients^36^. In the present study, peptides derived from COL1A1, COL1A2 were significantly increased 1-h after appliance placement, suggesting that these proteins display high susceptibility to proteolysis during OTM. This is supported by our protease activity predictions, which linked with the increased levels of detected collagen-derived peptides suggest that MMPs play roles in the breakdown of collagen and ECM remodelling during OTM. MMP8 and MMP13 are members of the collagenase group and MMP9 is a member of the gelatinase group; these proteases are effective in degrading type I collagen and gelatin^8,36^ and our data demonstrated increased predicted activity of both. Moreover, our gelatin zymography results showed increased gelatinolytic activity for two bands identified at 72 and 62 kDa over time but was statistically significant only 1-week after appliance placement. Differences in methodology could justify the inability to detect gelatinolytic activity at T2 but possibly suggests that a peptidomic approach might provide a better tool to detect the activity of proteases. Additionally, the increase in statherin-derived peptides 1-h after appliance placement maybe because fixed orthodontic appliance placement involving acid-etching of the teeth results in demineralization, and statherin is known to be involved in calcium homeostasis and teeth remineralization^25,35^. It also has a strong affinity for the tooth surface and was identified previously in the pellicle formed on metallic brackets^39^.

There were no statistically significant changes in plaque and gingival indices after 1-h and 1-week of orthodontic force application compared to baseline levels, and linear regression results showed no statistically significant association between the levels of MMP8, MMP9 with plaque and gingival indices over-time. Therefore, since changes in the proteolytic activity were observed in this study 1-h and 1-week after orthodontic appliance placement, we may assume that these changes were induced by orthodontic force rather than by bacterial plaque or gingival inflammation, suggesting that orthodontic forces modulate the proteolytic activity in the periodontal tissues.

Limitations of this study include being a retrospective in design, and using a small sample size with the mass spectrometry and bioinformatics approaches. Therefore, prospective clinical trials with a larger sample size should be ideally conducted. Additionally, further research focusing on the detailed characterization of the role of each identified protease and its substrates may enhance our understanding of the biology of OTM and possibly reveal novel biomarkers associated with OTM.

In conclusion, the profile and activity pattern of proteases responsible for salivary peptide generation have been mapped and susceptible protein targets have been identified during the alignment stage of fixed appliance orthodontic treatment. The proteases detected in WMS showed changes over time, with most MMPs and proteases associated with inflammation showing increases as early as 1-h after appliance placement, supported by increased collagen-derived peptides levels. Protease prediction from peptidome data demonstrates a potential tool for identifying and discriminating between different phases of OTM.

## Materials and methods

### Study design and participants

This retrospective longitudinal study evaluated WMS from 16 participants during the alignment phase of orthodontic treatment with fixed appliances. Participant inclusion was based upon the following criteria: undergoing fixed appliance orthodontic treatment (with or without tooth extractions); mandibular arch incisor irregularity of 4–12 mm; 12–18 years old at treatment-start; medically fit and healthy; taking no prescription medication and normal-weight body mass index.

Ethical approval was obtained from the United Kingdom National Research Ethics Service, NRES Committee foundation (14/LO/0769), and written informed consent was received from all parents, guardians, and children before sample collection. All methods were performed in accordance with the approved guidelines and regulations. All participants had their fixed appliances (Victory-APC 0.022-inch brackets, MBT prescription; 3M-Unitek) placed in the Department of Orthodontics, Faculty of Dentistry, Oral & Craniofacial Sciences, King’s College London (Guy’s and St Thomas; NHS Foundation Trust). A specific arch wire sequence was followed (0.014-inch nickel titanium; 0.018-inch nickel titanium; 0.017 × 0.025-inch nickel titanium and 0.019 × 0.025-inch stainless steel) and participants were seen every 6 weeks. Participants were followed up to completion of alignment between January 2015 and June 2016.

Sample size calculation was based on a previous study investigating time-dependent changes in salivary levels of MMP8 and MMP9 during orthodontic treatment. In this study, differences in levels of salivary biomarkers between different time-points were identified with a mean effect size of 0.87^40^. Using G*Power 3.1.9.7 software^41^, a sample size of 13 was estimated to be sufficient to detect a significant difference in salivary biomarker levels between the different time-points (assuming a power of 80% and a significance level of 5%). A sample size of 16 was used to compensate for power underestimation between the biomarkers.

### WMS collection and processing

Unstimulated WMS was collected at 4 time-points: (T1) start-of-treatment; (T2) 1-h and (T3) 1-week following fixed appliance placement; and (T4) completion of alignment (0.019 × 0.025-inch stainless steel rectangular archwire placed in the lower arch). WMS samples were centrifuged at 9200×*g* for 5-min, aliquoted, labelled and stored at − 80 °C. Samples were defrosted on ice and total protein concentration measured using a Bicinchoninic Acid (BCA) Protein Assay (Thermo-Scientific). For all participants, plaque levels and gingival health were measured at T1, T3 and T4 using established validated indices. The thickness of dental plaque adjacent to the gingival margin was measured using Silness and Löe criteria^42^, where a score from 0 to 3 is ascribed to each of the four surfaces of the tooth; these scores are then added and divided by four to provide the plaque index for each tooth. The plaque index for the individual is then calculated by adding the individual scores from each tooth and dividing them by the number of teeth examined. The gingival index was assessed using the same approach following Löe and Silness criteria^43^.

### Separation of naturally occurring peptides from WMS

Naturally-occurring peptides were collected from WMS of 5 participants at each time-point (20 samples). Ten kDa cut-off spin-filters (Merck-Millipore) were washed and conditioned with 500 µL 10 mM ammonium bicarbonate solution by centrifugation at 4000 rpm for 25-min. Spin-filters were loaded with 1 mL WMS, centrifuged at 4000 rpm for 25-min with the resulting filtrate peptides collected and sent for mass spectrometry (Cambridge Institute for Medical Research Proteomics Centre, UK).

### Mass spectrometry

A 100 µL WMS peptide sample was dried-down using a Savant SpeedVac Concentrator (Thermo-Scientific), solubilized in a 50 µL loading-solvent (3% acetonitrile; 0.1% trifluoroacetic acid) and 1 µL analysed by LC-MSMS using a Q-Exactive-Plus coupled to an RSLCnano3000 (Thermo-Scientific). Peptides were resolved on a 50 cm EASY-spray column (Thermo-Scientific) using a gradient rising from 3 to 40% solvent B (80% MeCN, 0.1% formic acid) by 90-min. MS-spectra were acquired at 70,000 (fwhm) between m/z 400 and 1500. Filtrate peptides were analysed by LC-MSMS using a Q-Exactive-Plus coupled to an RSLCnano3000 (Thermo-Scientific). Peptides were resolved on a 50 cm EASY-spray column (Thermo-Scientific) using a gradient rising from 10 to 40% solvent B (80% MeCN, 0.1% formic acid) by 42-min. The S-lens FR level was set to 50. MS-spectra were acquired at 70,000 (fwhm) between m/z 200 and 2000. Data were processed using PEAKS Studio (version X, Bioinformatics Solutions) with the following parameters: no enzyme; human database (UniProt reference proteome downloaded 18 Dec 2018 containing 21,066 proteins) or bacterial database (Uniprot proteome downloaded 5 Apr 2019 containing 161,286 proteins) with additional contaminant database (containing 246 common contaminants); oxidation (M), carbamidomethylation (C) as variable modifications at the PEAKS-DB stage, LFQ was carried out using PEAKS-LFQ using normalization by total protein intensity. Protein and protein-peptide information were exported from PEAKS-Studio.

### Zymography

Samples were analysed using 10% zymogram gelatin gel electrophoresis. Equal amounts of non-reducing Tris–Glycine SDS sample buffer (2×) and samples (10 µg) were loaded into the gel wells and run at 125v constant for 90-min with 10× Tris–Glycine SDS running. The gel was then placed in a zymogram renaturing buffer for 30-min, zymogram developing buffer (Life Technologies) for another 30-min (later replaced with a fresh developing buffer) and the gel incubated overnight at room temperature. Thereafter, the gel was stained with Coomassie Brilliant Blue and de-stained. Protease digestion appeared as transparent bands against a darkly stained blue background. Zymogram gel was scanned with ChemiDoc™ MP Imaging System (BioRad). Densitometric analysis was performed with Image J^44^.

### Enzyme-linked immunosorbent assay

Enzyme-linked immunosorbent assay (ELISA) (R&D Systems) was used to assess human total MMP8 and MMP9 (active and pro-active forms).

### Bioinformatic analysis

Protease prediction was carried out using the Proteasix tool in function of the peptides identified by mass spectrometry. Proteasix is an open-source peptide-centric tool that can be used to predict in silico the proteases involved in naturally occurring peptide generation and returns all possible proteases at a cleavage site (http://www.proteasix.org)^17^. Jvenn (http://bioinfo.genotoul.fr/jvenn/example.html) is an online Venn diagram tool used to find unique proteins at each time-point as well as those common to T1, T2, T3, T4.

### Statistical analysis

Data were tested for normality using Shapiro–Wilk and Kolmogorov–Smirnov tests. Repeated measures ANOVA was used to analyse the normally-distributed data followed by correction for multiple testing using Dunnett’s multiple-comparisons test. Friedman test was used to analyse the non-normally distributed data followed by correction for multiple testing using Bonferroni. Regression-analysis was performed of the outcome (MMP8, MMP9-levels), explorative factor (plaque index or gingival index) and variation through time-points. Statistical analysis was performed using GraphPad Prism version 9.0 (GraphPad Software). The differences were considered statistically significant if p < 0.05.

## Supplementary Information

Supplementary Information.

## Data Availability

The datasets generated during and/or analysed during the current study are available from the corresponding author on request.
