# Supplementary material for: Salivary peptidome analysis and protease prediction during orthodontic treatment with fixed appliances
Source: Sci Rep. 2023 Jan 12;13:677. doi: 10.1038/s41598-022-26969-3 (PMC9837200; doi:10.1038/s41598-022-26969-3)
Supplement: Supplementary file 1 — Supplementary Information. [file 41598_2022_26969_MOESM1_ESM.pdf]

Salivary peptidome analysis and protease prediction during orthodontic treatment with fixed appliances

Fidaa Wazwaz [1,*], Hayder Saloom [1,3], Jack W Houghton [4], Martyn T. Cobourne [1], Guy H. Carpenter [2]

[1] Department of Orthodontics, Faculty of Dentistry, Oral & Craniofacial Sciences, Centre for Craniofacial Development & Regeneration, King's College London, UK

[2] Salivary Biology, Centre for Host-Microbiome Interactions, Faculty of Dentistry, Oral & Craniofacial Sciences, King's College London, UK

[3] Orthodontic Department, College of Dentistry, University of Baghdad, Iraq

[4] Department of Chemistry, Molecular Sciences Research Hub, Imperial College London, UK

*Correspondence to: Fidaa Wazwaz, Department of Orthodontics, Faculty of Dentistry, Oral & Craniofacial Sciences, Centre for Craniofacial Development & Regeneration, King's College London, Guy’s Hospital, London SE1 9RT, UK. E-mail: fidaa.wazwaz@kcl.ac.uk.co.uk

SUPPLEMENTARY MATERIAL

Appendix Table 1 The top 15 common proteins and percentage of peptides for each identified at each time-point.

Protein ID	Protein Name	T1					
		P1	P2	P3	P4	P5	Mean
PIGR_HUMAN	Polymeric immunoglobulin receptor	0.42	0.86	0.34	1.60	0.68	0.78
PRP1_HUMAN	Basic salivary proline-rich protein 1	23.55	20.80	15.54	30.19	24.30	22.88
PRB2_HUMAN	Basic salivary proline-rich protein 2	19.99	19.99	18.51	27.45	22.38	21.66
PRB3_HUMAN	Basic salivary proline-rich protein 3	5.94	7.22	9.49	4.34	8.68	7.13
PRB4_HUMAN	Basic salivary proline-rich protein 4	5.48	8.09	12.63	8.49	11.72	9.28
PRPC_HUMAN	Salivary acidic proline-rich phosphoprotein 1/2	8.74	11.95	11.83	14.8	9.73	11.41
SMR3B_HUMAN	Submaxillary gland androgen-regulated protein 3B	8.16	4.68	5.94	4.22	6.51	5.90
STAT_HUMAN	Statherin	6.27	4.48	2.29	1.32	3.16	3.50
HIS1_HUMAN	Histatin-1	3.47	1.53	3.14	1.70	2.98	2.56
HIS3_HUMAN	Histatin-3	1.25	0.81	1.77	2.55	1.12	1.50
PRR27_HUMAN	Proline-rich protein 27	0.42	0.20	0.17	0.19	0.12	0.22
PROL4_HUMAN	Proline-rich protein 4	0.71	0.76	0.23	0.0	0.06	0.35
CO1A1_HUMAN	Collagen alpha-1(I)	0.04	0.25	0	0.38	0.19	0.17
CO2A1_HUMAN	Collagen alpha-1(II)	0.18	0.15	0.11	0.19	0.12	0.15
TR_HUMAN	Uncharacterized protein OS	8.36	11.55	12.06	0.09	0	6.41
**Protein ID**	**Protein Name**	**T2**					
		P1	P2	P3	P4	P5	Mean
PIGR_HUMAN	Polymeric immunoglobulin receptor	1.11	1.19	0.7	1.47	1.03	1.10

PRP1_HUMAN	Basic salivary proline-rich protein 1	18.82	19.32	14.68	22.03	25.59	20.09
PRB2_HUMAN	Basic salivary proline-rich protein 2	16.34	18.16	16.42	22.11	21.96	19.00
PRB3_HUMAN	Basic salivary proline-rich protein 3	5.69	5.01	8.58	3.94	5.8	5.80
PRB4_HUMAN	Basic salivary proline-rich protein 4	3.21	4.98	10.26	6.87	7.69	6.60
PRPC_HUMAN	Salivary acidic proline-rich phosphoprotein 1/2	9.83	9.8	9.07	8.79	8.03	9.10
SMR3B_HUMAN	Submaxillary gland androgen-regulated protein 3B	7.51	6.64	5.82	4.69	4.22	5.78
STAT_HUMAN	Statherin	7.57	7.64	6.77	7.04	6.21	7.05
HIS1_HUMAN	Histatin-1	3.21	2.35	4.11	2.43	1.78	2.78
HIS3_HUMAN	Histatin-3	0.22	0.28	0.98	1.17	0.41	0.61
PRR27_HUMAN	Proline-rich protein 27	1.7	1.06	0.98	0.42	0.48	0.93
PROL4_HUMAN	Proline-rich protein 4	1.48	1.6	1.44	0.54	0.38	1.09
CO1A1_HUMAN	Collagen alpha-1(I)	1.11	1.1	0.77	0.63	0.41	0.75
CO2A1_HUMAN	Collagen alpha-1(II)	0.56	0.53	0.58	0.88	0.45	0.6
TR_HUMAN	Uncharacterized protein OS	9.3	9.46	9.59	8.46	7.75	8.91
**Protein ID**	**Protein Name**	**T3**					
		P1	P2	P3	P4	P5	Mean
PIGR_HUMAN	Polymeric immunoglobulin receptor	1.78	1.75	0.77	1.64	1.18	1.424
PRP1_HUMAN	Basic salivary proline-rich protein 1	14.57	18.1	17.78	22.2	24.79	19.49
PRB2_HUMAN	Basic salivary proline-rich protein 2	12.75	18.22	19.28	21.15	22.06	18.69
PRB3_HUMAN	Basic salivary proline-rich protein 3	8.12	6.39	7.31	5.62	4.66	6.42
PRB4_HUMAN	Basic salivary proline-rich protein 4	6.10	6.36	8.7	8.66	7.21	7.41
PRPC_HUMAN	Salivary acidic proline-rich phosphoprotein 1/2	14.36	11.93	9.85	11.67	8.17	11.20
SMR3B_HUMAN	Submaxillary gland androgen-regulated protein 3B	5.18	5.73	4.14	3.59	4.85	4.70

STAT_HUMAN	Statherin	5.38	7.04	6.19	4.53	5.97	5.82
HIS1_HUMAN	Histatin-1	1.34	1.75	3.38	1.76	0.9	1.83
HIS3_HUMAN	Histatin-3	0.1	0.34	0.0	1.13	0.28	0.37
PRR27_HUMAN	Proline-rich protein 27	1.02	0.41	0.94	0.55	0.4	0.66
PROL4_HUMAN	Proline-rich protein 4	0.82	1.0	1.15	0.43	0.53	0.79
CO1A1_HUMAN	Collagen alpha-1(I)	0.21	0.44	0.87	0.94	0.37	0.57
CO2A1_HUMAN	Collagen alpha-1(II)	0.34	0.31	0.28	0.2	0.19	0.26
TR_HUMAN	Uncharacterized protein OS	13.54	11.58	10.16	11.39	7.92	10.92
**Protein ID**	**Protein Name**	**T4**					
		P1	P2	P3	P4	P5	Mean
PIGR_HUMAN	Polymeric immunoglobulin receptor	0.83	1.11	0.5	1.91	0.67	1.00
PRP1_HUMAN	Basic salivary proline-rich protein 1	23.28	20.08	14.28	24.48	24.36	21.30
PRB2_HUMAN	Basic salivary proline-rich protein 2	19.93	19.36	15.99	22.93	21.29	19.90
PRB3_HUMAN	Basic salivary proline-rich protein 3	5.74	12.43	9.91	6.01	8.83	8.58
PRB4_HUMAN	Basic salivary proline-rich protein 4	5.96	12.69	12.56	7.55	11.47	10.05
PRPC_HUMAN	Salivary acidic proline-rich phosphoprotein 1/2	10.18	14.72	12.4	19.84	11.23	13.67
SMR3B_HUMAN	Submaxillary gland androgen-regulated protein 3B	6.92	5.10	4.54	3.73	6.44	5.35
STAT_HUMAN	Statherin	4.44	3.92	3.43	1.36	3.25	3.28
HIS1_HUMAN	Histatin-1	2.31	0.78	3.21	1.73	2.82	2.17
HIS3_HUMAN	Histatin-3	0.83	0.0	1.72	2.91	0.43	1.18
PRR27_HUMAN	Proline-rich protein 27	0.65	0.46	0.22	0.0	0.18	0.30
PROL4_HUMAN	Proline-rich protein 4	0.83	1.24	0.39	0.27	0.12	0.57
CO1A1_HUMAN	Collagen alpha-1(I)	0.13	0.13	0.22	0.27	0.31	0.21
CO2A1_HUMAN	Collagen alpha-1(II)	0	0.2	0.11	0.27	0.18	0.15

TR_HUMAN	Uncharacterized protein OS	9.88	0.0	12.73	0.0	0.0	4.52

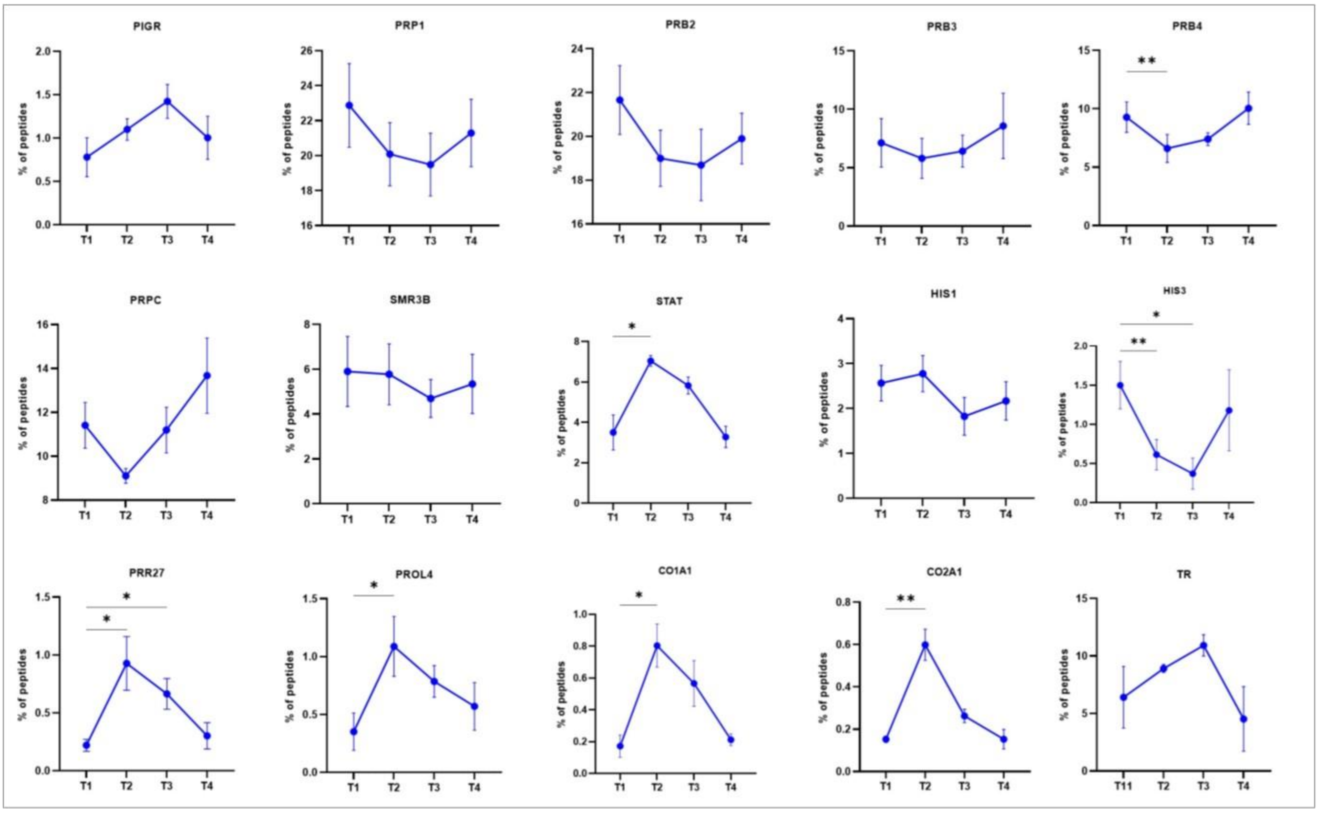

Appendix Figure 1 Graphs showing the percentage of peptides for each of the common proteins identified in unstimulated WMS at T1-T4. T1, baseline (before placement of fixed appliances); T2, 1 hour after placement of fixed appliances; T3, 1 week after placement of fixed appliances; T4, completion of alignment. Data were analysed by repeated measures ANOVA; $p<0.05$ *; $p<0.01$ **.

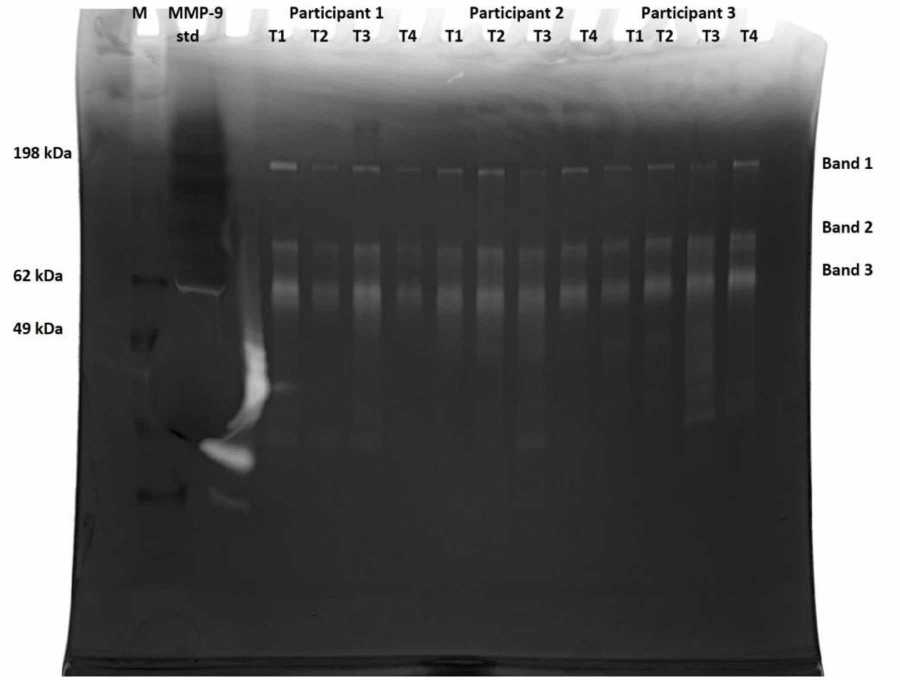

Appendix Figure 2 Representative example of Coomassie-stained zymogram gel demonstrating bands with gelatinolytic activity in unstimulated whole mouth saliva at three different molecular weights.
